# Supplementary material for: The Impact of Non-optimum Ambient Temperature on Years of Life Lost: A Multi-county Observational Study in Hunan, China
Source: Int J Environ Res Public Health. 2020 Apr 14;17(8):2699. doi: 10.3390/ijerph17082699 (PMC7215980; doi:10.3390/ijerph17082699)
Supplement: Supplementary file 1 [file ijerph-17-02699-s001.pdf]

Table S1. The life table of Hunan province.

Age group	Life expectancy		
	**Total population**	**Male**	**Female**
0	78.60	76.43	81.04
1–4	77.81	75.62	80.25
5–9	74.04	71.87	76.46
10–14	69.15	67.01	71.55
15–19	64.26	62.13	66.62
20–24	59.37	57.28	61.70
25–29	54.51	52.45	56.79
30–34	49.66	47.66	51.89
35–39	44.85	42.91	47.02
40–44	40.11	38.23	42.20
45–49	35.44	33.65	37.44
50–54	30.85	29.15	32.74
55–59	26.51	24.93	28.24
60–64	22.25	20.81	23.81
65–69	18.23	16.93	19.59
70–74	14.59	13.45	15.73
75–79	11.47	10.50	12.37
80–84	8.87	8.10	9.51
85–89	6.85	6.20	7.30
90–94	5.32	4.89	5.58
95–99	4.24	4.00	4.37
100+	2.07	1.40	2.45

Table S2. Sensitivity analysis was used to estimate the association between ambient temperatures and YLL for non-accidental disease death.

Lag period	Df for year	Df for relative humidity	AIC
14	6	3	13363.84
14	6	4	13365.28
14	6	5	13366.27
14	7	3	13352.39
14	7	4	13353.51
14	7	5	13354.30
14	8	3	13367.99
14	8	4	13369.22
14	8	5	13370.20
21	6	3	13294.24
21	6	4	13295.76
21	6	5	13297.12
**21**	**7**	**3**	**13283.90**
21	7	4	13285.19
21	7	5	13286.27
21	8	3	13294.35
21	8	4	13295.78
21	8	5	13297.04

Df: degree of freedom; AIC: Akaike's Information Criterion.
